# 4-(Dimethyl­amino)­pyridinium tetra­chloridoferrate(III)

**DOI:** 10.1107/S160053681300603X

**Published:** 2013-03-09

**Authors:** Amina Khadri, Rafika Bouchene, Sofiane Bouacida, Hocine Merazig, Thierry Roisnel

**Affiliations:** a Laboratoire de Chimie Appliquée et Technologie des Matériaux LCATM, Université Oum El Bouaghi, Algeria; b Département Sciences de la Matière, Faculté des Sciences Exactes et Sciences de la Nature et de la Vie, Université Oum El Bouaghi, Algeria; c Unité de Recherche de Chimie de l’Environnement et Moléculaire Structurale, CHEMS, Faculté des Sciences Exactes, Université Mentouri Constantine 25000, Algeria; d Centre de Diffractométrie X, UMR 6226 CNRS Unité Sciences Chimiques de Rennes, Université de Rennes I, 263 Avenue du Général Leclerc, 35042 Rennes, France

## Abstract

The title salt, (C_7_H_11_N_2_)[FeCl_4_], consists of one essentially planar (the r.m.s. deviation for all non-H atoms being 0.004 Å) 4-(dimethyl­amino)­pyridinium cation and a tetra­hedral tetra­chloridoferrate(III) anion. The cations and anions are arranged in layers parallel to (010). Besides electrostatic inter­actions, the crystal packing features N—H⋯Cl and C—H⋯Cl hydrogen bonds between cations and anions, forming a three-dimensional network.

## Related literature

For background to hybrid compounds based on protonated substituted *N*-heterocyclic ligands, see: Bouacida (2008 ▶); Bouacida *et al.* (2007 ▶, 2009 ▶). For a related structure, see: Nenwa *et al.* (2010 ▶).
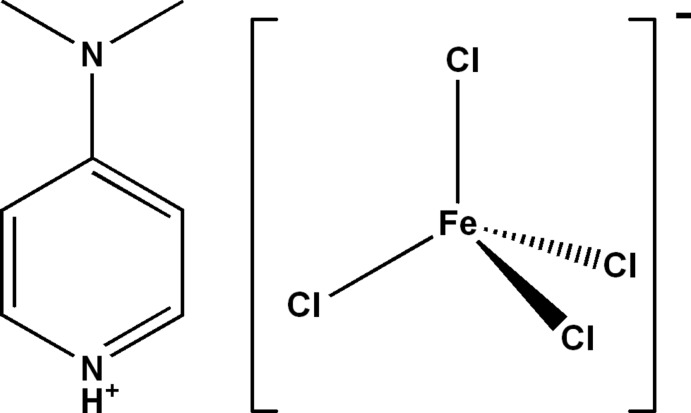


## Experimental

### 

#### Crystal data

(C_7_H_11_N_2_)[FeCl_4_]
*M* *_r_* = 320.83
Monoclinic, 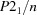

*a* = 9.0360 (2) Å
*b* = 14.0492 (5) Å
*c* = 10.2077 (3) Å
β = 98.7259 (9)°
*V* = 1280.85 (7) Å^3^
*Z* = 4
Mo *K*α radiation
μ = 1.98 mm^−1^
*T* = 100 K
0.17 × 0.12 × 0.04 mm

#### Data collection

Bruker APEXII CCD diffractometer
Absorption correction: multi-scan (*SADABS*; Bruker, 2011 ▶) *T*_min_ = 0.789, *T*_max_ = 0.924
11192 measured reflections
2925 independent reflections
2146 reflections with *I* > 2σ(*I*)
*R*_int_ = 0.041

#### Refinement

*R*[*F* ^2^ > 2σ(*F* ^2^)] = 0.034
*wR*(*F* ^2^) = 0.081
*S* = 1.03
2925 reflections
133 parameters
H atoms treated by a mixture of independent and constrained refinement
Δρ_max_ = 0.94 e Å^−3^
Δρ_min_ = −0.56 e Å^−3^

### 

Data collection: *APEX2* (Bruker, 2011 ▶); cell refinement: *SAINT* (Bruker, 2011 ▶); data reduction: *SAINT*; program(s) used to solve structure: *SIR2004* (Burla *et al.*, 2005 ▶); program(s) used to refine structure: *SHELXL97* (Sheldrick, 2008 ▶); molecular graphics: *ORTEP-3 for Windows* (Farrugia, 2012 ▶) and *DIAMOND* (Brandenburg & Berndt, 2001 ▶); software used to prepare material for publication: *WinGX* (Farrugia, 2012 ▶).

## Supplementary Material

Crystal structure: contains datablock(s) global, I. DOI: 10.1107/S160053681300603X/wm2729sup1.cif

Structure factors: contains datablock(s) I. DOI: 10.1107/S160053681300603X/wm2729Isup2.hkl

Additional supplementary materials:  crystallographic information; 3D view; checkCIF report

## Figures and Tables

**Table 1:** Hydrogen-bond geometry (Å, °)

*D*—H⋯*A*	*D*—H	H⋯*A*	*D*⋯*A*	*D*—H⋯*A*
N3—H3⋯Cl2^i^	0.79 (3)	2.60 (3)	3.369 (3)	165 (3)
C4—H4⋯Cl1^i^	0.95	2.74	3.604 (3)	152

Symmetry code: (i) 
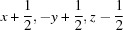
.

**Fig. 1.**
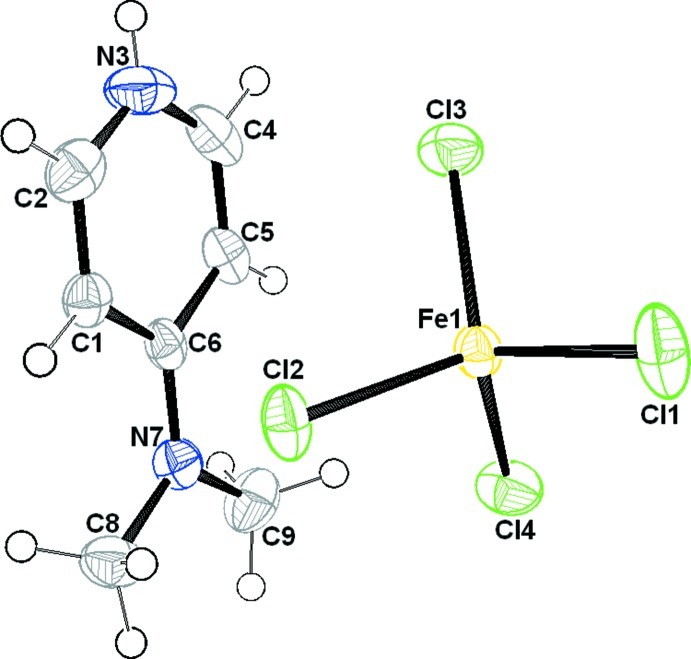
The asymmetric unit of (I) with the atom-labelling scheme. Displacement ellipsoids are drawn at the 50% probability level. H atoms are represented as small spheres of arbitrary radii.

**Fig. 2.**
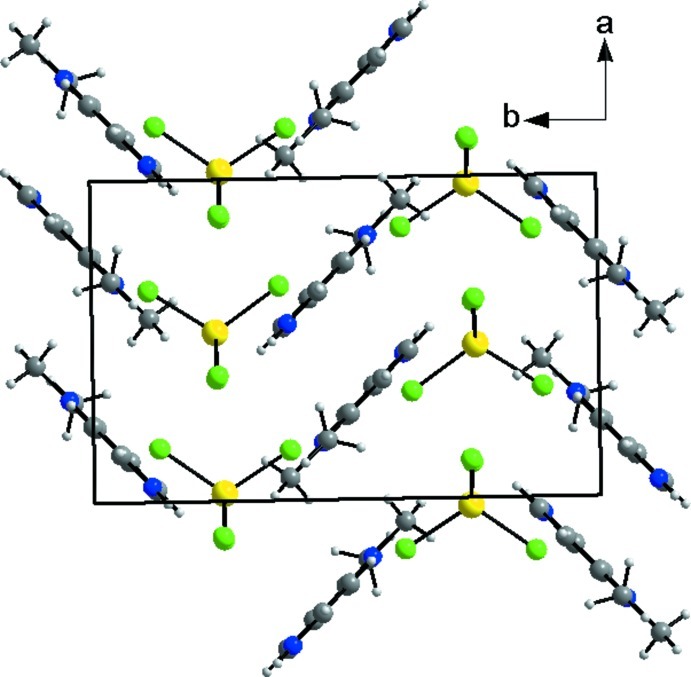
Packing diagram viewed along [001] showing alterning layers of 4-(dimethylamino)pyridinium cations and [FeCl4] tetrahedraparallel to (010).

**Fig. 3.**
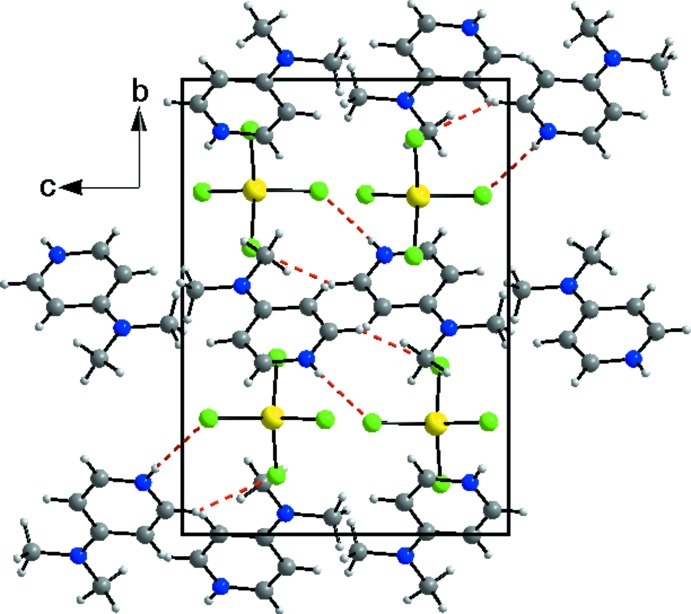
Packing diagram viewed along [100] showing N—H···Cl and C—H···Cl hydrogen-bonding interactions as dashed lines.

## supplementary crystallographic information

### Comment

In the course of previuos studies on intermolecular hydrogen-bonding interactions in transition metal hybrid complexes with *N*-heterocyclic ligands (Bouacida, 2008; Bouacida *et al.*, 2007, 2009), we report here on synthesis and structural characterization of a new organic/inorganic hybrid, involving Fe(III) and 4-(dimethylamino)pyridine, (C_7_H_11_N_2_)^+^^.^[FeCl_4_]^-^, (I). The molecular geometry of the constituents and the atom-numbering scheme of (I) are shown in Fig. 1.

The asymmetric unit of (I) consists of tetrahedral [FeCl_4_]^-^ anions and one protoned 4-(dimethylamino)pyridine cation that is essentially planar; its r.m.s. deviation for all non-H atoms is 0.0043 Å, with a maximum deviation from the mean plane of -0.0094 (2) Å for the C4 atom. The packing of the ionic entities is realized by alternating layers of cations and anions parallel to (010) whereby the dimethylaminopyridinium molecules are oriented in a zig-zag fashion parallel to the (210) and (210) planes, respectively (Fig. 2). The crystal packing is stabilized by N—H···Cl and C—H···Cl hydrogen bonds involving the chloride atoms of the anions as acceptors (Table 1, Fig. 3). All these interactions link the layers together, forming a three-dimensional network and reinforcing the cohesion of the ionic structure.

A similar complex with a 4-(dimethylamino)pyridinium cation but a different metal-based anion, *viz.* (C_7_H_11_N_2_)^+^[Cr(C_2_O_4_)_2_(H_2_O)_2_]^-^, has been reported recently (Nenwa *et al.*, 2010).

### Experimental

4-(dimethylamino)pyridine and iron(III) chloride hexahydrate were mixed in an equimolar ratio in acidified water (HCl, 37%_wt_). The solution was kept at room temperature for ten days after which crystals suitable for X-ray diffraction could be isolated.

### Refinement

H atoms were localized from Fourier maps but introduced in calculated positions and treated as riding on their parent C atoms with C—H = 0.98 Å (methyl) or C—H = 0.95 Å (aromatic), and with *U*_iso_(H) = 1.2 *U*_eq_(C_aryl_)and *U*_iso_(H) = 1.5 *U*_eq_(C_methyl_). H3 attached to the pyridinium N atom was refined without constraints.

### Crystal data

**Table:**

(C_7_H_11_N_2_)[FeCl_4_]	*F*(000) = 644
*M**_r_* = 320.83	*D*_x_ = 1.664 Mg m^−^^3^
Monoclinic, *P*2_1_/*n*	Mo *K*α radiation, λ = 0.71073 Å
Hall symbol: -P 2yn	Cell parameters from 2278 reflections
*a* = 9.0360 (2) Å	θ = 2.9–26.5°
*b* = 14.0492 (5) Å	µ = 1.98 mm^−^^1^
*c* = 10.2077 (3) Å	*T* = 100 K
β = 98.7259 (9)°	Plate, orange
*V* = 1280.85 (7) Å^3^	0.17 × 0.12 × 0.04 mm
*Z* = 4	

### Data collection

**Table:**

Bruker APEXII CCD diffractometer	2925 independent reflections
Radiation source: sealed tube	2146 reflections with *I* > 2σ(*I*)
Graphite monochromator	*R*_int_ = 0.041
φ and ω scans	θ_max_ = 27.5°, θ_min_ = 2.5°
Absorption correction: multi-scan (*SADABS*; Bruker, 2011)	*h* = −8→11
*T*_min_ = 0.789, *T*_max_ = 0.924	*k* = −18→18
11192 measured reflections	*l* = −13→13

### Refinement

**Table:**

Refinement on *F*^2^	Primary atom site location: structure-invariant direct methods
Least-squares matrix: full	Secondary atom site location: difference Fourier map
*R*[*F*^2^ > 2σ(*F*^2^)] = 0.034	Hydrogen site location: inferred from neighbouring sites
*wR*(*F*^2^) = 0.081	H atoms treated by a mixture of independent and constrained refinement
*S* = 1.03	*w* = 1/[σ^2^(*F*_o_^2^) + (0.0258*P*)^2^ + 0.6542*P*] where *P* = (*F*_o_^2^ + 2*F*_c_^2^)/3
2925 reflections	(Δ/σ)_max_ = 0.001
133 parameters	Δρ_max_ = 0.94 e Å^−^^3^
0 restraints	Δρ_min_ = −0.56 e Å^−^^3^

### Special details

**Table:**

Geometry. All e.s.d.'s (except the e.s.d. in the dihedral angle between two l.s. planes) are estimated using the full covariance matrix. The cell e.s.d.'s are taken into account individually in the estimation of e.s.d.'s in distances, angles and torsion angles; correlations between e.s.d.'s in cell parameters are only used when they are defined by crystal symmetry. An approximate (isotropic) treatment of cell e.s.d.'s is used for estimating e.s.d.'s involving l.s. planes.
Refinement. Refinement of *F*^2^ against ALL reflections. The weighted *R*-factor *wR* and goodness of fit *S* are based on *F*^2^, conventional *R*-factors *R* are based on *F*, with *F* set to zero for negative *F*^2^. The threshold expression of *F*^2^ > σ(*F*^2^) is used only for calculating *R*-factors(gt) *etc*. and is not relevant to the choice of reflections for refinement. *R*-factors based on *F*^2^ are statistically about twice as large as those based on *F*, and *R*- factors based on ALL data will be even larger.

### Fractional atomic coordinates and isotropic or equivalent isotropic displacement parameters (Å^2^)

**Table:**

	*x*	*y*	*z*	*U*_iso_*/*U*_eq_	
Fe1	−0.02427 (4)	0.25669 (2)	0.72252 (3)	0.02371 (11)	
Cl1	−0.16650 (8)	0.13001 (5)	0.71136 (7)	0.0449 (2)	
Cl2	0.11905 (7)	0.25489 (4)	0.91821 (6)	0.03315 (17)	
Cl3	0.11063 (8)	0.25245 (5)	0.56193 (7)	0.03676 (18)	
Cl4	−0.15646 (8)	0.38804 (5)	0.70930 (7)	0.03842 (18)	
C1	0.3730 (3)	0.42873 (17)	0.8197 (3)	0.0281 (6)	
H1	0.381	0.4214	0.913	0.034*	
C2	0.4618 (3)	0.37690 (19)	0.7516 (3)	0.0374 (7)	
H2	0.5322	0.3338	0.7976	0.045*	
N3	0.4513 (3)	0.38586 (19)	0.6189 (3)	0.0445 (7)	
H3	0.506 (4)	0.355 (2)	0.582 (3)	0.059 (11)*	
C4	0.3518 (3)	0.4461 (2)	0.5506 (3)	0.0417 (7)	
H4	0.3453	0.4501	0.457	0.05*	
C5	0.2614 (3)	0.50068 (19)	0.6130 (3)	0.0331 (6)	
H5	0.1935	0.5436	0.5635	0.04*	
C6	0.2677 (3)	0.49407 (17)	0.7526 (2)	0.0260 (6)	
N7	0.1790 (2)	0.54643 (15)	0.8167 (2)	0.0305 (5)	
C8	0.1866 (3)	0.5381 (2)	0.9603 (3)	0.0407 (7)	
H8A	0.1606	0.4731	0.9829	0.061*	
H8B	0.116	0.5829	0.9908	0.061*	
H8C	0.2884	0.5529	1.0036	0.061*	
C9	0.0706 (3)	0.6131 (2)	0.7463 (3)	0.0448 (8)	
H9A	0.1236	0.6617	0.7024	0.067*	
H9B	0.0143	0.6437	0.8095	0.067*	
H9C	0.0013	0.5786	0.6797	0.067*	

### Atomic displacement parameters (Å^2^)

**Table:**

	*U*^11^	*U*^22^	*U*^33^	*U*^12^	*U*^13^	*U*^23^
Fe1	0.0239 (2)	0.0260 (2)	0.02024 (19)	−0.00208 (14)	0.00016 (14)	0.00140 (15)
Cl1	0.0505 (4)	0.0485 (4)	0.0315 (4)	−0.0268 (3)	−0.0077 (3)	0.0074 (3)
Cl2	0.0327 (3)	0.0370 (4)	0.0261 (3)	−0.0089 (3)	−0.0072 (3)	0.0047 (3)
Cl3	0.0380 (4)	0.0417 (4)	0.0330 (4)	−0.0003 (3)	0.0131 (3)	−0.0032 (3)
Cl4	0.0419 (4)	0.0406 (4)	0.0322 (4)	0.0157 (3)	0.0035 (3)	0.0016 (3)
C1	0.0269 (13)	0.0283 (14)	0.0287 (14)	−0.0048 (11)	0.0035 (11)	0.0028 (11)
C2	0.0331 (15)	0.0305 (15)	0.050 (2)	−0.0057 (12)	0.0092 (13)	0.0032 (13)
N3	0.0481 (16)	0.0367 (15)	0.0554 (18)	−0.0106 (12)	0.0295 (14)	−0.0140 (13)
C4	0.0578 (19)	0.0419 (17)	0.0266 (16)	−0.0218 (15)	0.0103 (14)	−0.0071 (13)
C5	0.0404 (16)	0.0321 (15)	0.0255 (14)	−0.0088 (12)	0.0002 (12)	−0.0008 (11)
C6	0.0276 (14)	0.0264 (13)	0.0235 (13)	−0.0107 (11)	0.0022 (11)	−0.0001 (10)
N7	0.0314 (12)	0.0300 (12)	0.0299 (13)	−0.0015 (9)	0.0036 (10)	−0.0003 (10)
C8	0.0467 (17)	0.0427 (17)	0.0360 (17)	−0.0037 (14)	0.0167 (14)	−0.0030 (13)
C9	0.0347 (16)	0.0370 (17)	0.061 (2)	0.0049 (13)	0.0025 (14)	0.0062 (15)

### Geometric parameters (Å, º)

**Table:**

Fe1—Cl3	2.1870 (7)	C4—H4	0.95
Fe1—Cl1	2.1882 (7)	C5—C6	1.420 (3)
Fe1—Cl4	2.1912 (7)	C5—H5	0.95
Fe1—Cl2	2.2097 (7)	C6—N7	1.331 (3)
C1—C2	1.351 (4)	N7—C8	1.462 (3)
C1—C6	1.421 (3)	N7—C9	1.463 (3)
C1—H1	0.95	C8—H8A	0.98
C2—N3	1.350 (4)	C8—H8B	0.98
C2—H2	0.95	C8—H8C	0.98
N3—C4	1.349 (4)	C9—H9A	0.98
N3—H3	0.79 (3)	C9—H9B	0.98
C4—C5	1.348 (4)	C9—H9C	0.98
			
Cl3—Fe1—Cl1	109.18 (3)	C6—C5—H5	119.9
Cl3—Fe1—Cl4	109.72 (3)	N7—C6—C5	121.4 (2)
Cl1—Fe1—Cl4	111.80 (3)	N7—C6—C1	122.0 (2)
Cl3—Fe1—Cl2	111.12 (3)	C5—C6—C1	116.6 (2)
Cl1—Fe1—Cl2	107.27 (3)	C6—N7—C8	120.6 (2)
Cl4—Fe1—Cl2	107.73 (3)	C6—N7—C9	121.4 (2)
C2—C1—C6	120.4 (3)	C8—N7—C9	118.0 (2)
C2—C1—H1	119.8	N7—C8—H8A	109.5
C6—C1—H1	119.8	N7—C8—H8B	109.5
N3—C2—C1	120.6 (3)	H8A—C8—H8B	109.5
N3—C2—H2	119.7	N7—C8—H8C	109.5
C1—C2—H2	119.7	H8A—C8—H8C	109.5
C4—N3—C2	121.1 (3)	H8B—C8—H8C	109.5
C4—N3—H3	121 (3)	N7—C9—H9A	109.5
C2—N3—H3	118 (3)	N7—C9—H9B	109.5
C5—C4—N3	121.1 (3)	H9A—C9—H9B	109.5
C5—C4—H4	119.4	N7—C9—H9C	109.5
N3—C4—H4	119.4	H9A—C9—H9C	109.5
C4—C5—C6	120.1 (3)	H9B—C9—H9C	109.5
C4—C5—H5	119.9		
			
C6—C1—C2—N3	−0.4 (4)	C2—C1—C6—N7	−179.8 (2)
C1—C2—N3—C4	−0.4 (4)	C2—C1—C6—C5	0.4 (3)
C2—N3—C4—C5	1.3 (4)	C5—C6—N7—C8	179.6 (2)
N3—C4—C5—C6	−1.3 (4)	C1—C6—N7—C8	−0.2 (4)
C4—C5—C6—N7	−179.4 (2)	C5—C6—N7—C9	0.1 (4)
C4—C5—C6—C1	0.4 (3)	C1—C6—N7—C9	−179.7 (2)

### Hydrogen-bond geometry (Å, º)

**Table:**

*D*—H···*A*	*D*—H	H···*A*	*D*···*A*	*D*—H···*A*
N3—H3···Cl2^i^	0.79 (3)	2.60 (3)	3.369 (3)	165 (3)
C4—H4···Cl1^i^	0.95	2.74	3.604 (3)	152

Symmetry code: (i) *x*+1/2, −*y*+1/2, *z*−1/2.
